# Living with diabetes—Development of learning patterns over a 3-year period

**DOI:** 10.3402/qhw.v9.24375

**Published:** 2014-07-15

**Authors:** Åsa Kneck, Ingegerd Fagerberg, Lars E. Eriksson, Berit Lundman

**Affiliations:** 1 Department of Health Care Sciences, Ersta Sköndal University College, Stockholm, Sweden; 2 Division of Nursing, Department of Neurolobiology, Care Sciences and Society, Karolinska Institutet, Solna, Sweden; 3 Department of Infectious Diseases, Karolinska University Hospital, Stockholm, Sweden; 4 School of Health Sciences, City University London, London, United Kingdom; 5 Department of Nursing, Umeå Universitet, Umeå, Sweden

**Keywords:** Activities of daily living, chronic illness, knowledge, problem-solving self-care, self-management

## Abstract

**Background:**

Learning involves acquiring new knowledge and skills, and changing our ways of thinking, acting, and feeling. Learning in relation to living with diabetes is a lifelong process where there is limited knowledge of how it is experienced and established over time. It was considered important to explore how learning was developed over time for persons living with diabetes.

**Aim:**

The aim of the study was to identify patterns in learning when living with diabetes, from recently being diagnosed, and over a 3-year period.

**Materials and methods:**

A longitudinal qualitative descriptive design was used. Thirteen participants, with both type I and type II diabetes, were interviewed at three different occasions during a 3-year period. Qualitative content analysis was used in different steps in order to distinguish patterns.

**Findings:**

Five main patterns of learning were identified. Two of the patterns (I and II) were characterized by gradually becoming comfortable living with diabetes, whereas for one pattern (IV) living with diabetes became gradually more difficult. For pattern V living with diabetes was making only a limited impact on life, whereas for Pattern III there was a constant management of obstacles related to illness. The different patterns in the present study showed common and different ways of learning and using different learning strategies at different timespans.

**Conclusion:**

The present study showed that duration of illness is not of importance for how far a person has come in his own learning process. A person-centered care is needed to meet the different and changing needs of persons living with diabetes in relation to learning to live with a lifelong illness.

Learning involves acquiring new knowledge and skills and changes in the way a person thinks, acts, and feels, associated with personal growth and gaining experience (Jarvis, Holford, & Griffin, 2003). Schultz and Luckmann (1973) describe learning as making people better prepared to deal with life or to feel balanced between their personal capacity and the demands they face. In relation to chronic illness, learning has been described in terms of “taking action to create order” (Kralik, Koch, Price, & Howard, 2004) and in relation to diabetes as a “balancing act” (Paterson, Thorne, & Dewis, 1998). To meet, understand, and manage changes in relation to chronic illness has also been described in relation to transition (Kralik, Visentin, & Van Loon, 2006; Meleis, Sawyer, Im, Hilfinger Messias, & Schumacher, 2000), illness integration (Hörnsten, Jutterström, Audulv, & Lundman, 2011), adaption, adjustments and development (Paterson et al., 1998), and self-management (Kralik et al., 2004; McEwen, Baird, Pasvogel, & Gallegos, 2007). Illness integration, such as managing the consequences of chronic illness as part of a natural way of living, is often considered a successful learning process (Kralik et al., 2004; Whittemore, Chase, Mandle, & Roy, 2002).

To be active promotes learning (Knowles, 1990), and to be active in one’s own diabetes care and feel responsibility for decision-making has been found to be crucial for illness integration (Kralik et al., 2005; Paterson & Thorne, 2000), achievement of good blood glucose control (Vég, 2006), and being independent of health care (Govan et al., 2011; Hernandez, 1995; Price, 1993). However, illness integration as well as satisfying blood glucose control is neverless reached by all persons with diabetes (Bryant, Greenfield, Chrisholm, & Campbell, 2006; Coyle, Francis, & Champan, 2013; Moses et al., 2012; Perry, Steinbeck, Dunbabin, & Lowe, 2010), and unsatisfactory blood glucose control is associated with higher levels of “diabetes burden” (Wikblad, Smide, & Leksell, 2014).

Time and time span are elements of a learning process, as development of knowledge and skills are understood to occur over time (Meleis et al., 2000). Audulv, Norbergh, Asplund, and Hörnsten (2009) in line with Chapple and Rogers (2001) found that confidence in the ability to take care of oneself developed gradually over time. However, Riegel, Jaarsma, and Strömberg (2012) found that even years after living with diabetes some participants never came to terms with self-management. In the study by Skovlund and Peyrot (2005) a majority of participants reported problems of living with diabetes as common, years after diagnosis.

It is not well understood how people experience and respond to changes over time and how the duration of illness contributes to the learning process when living with diabetes (Kralik et al., 2006; Rasmussen, Ward, Jenkins, King, & Dunning, 2011). There is no consensus among researchers as to whether the learning process in relation to living with chronic illness is linear, shifting, or fluctuating (Kralik et al., 2006). Persson, Winkvist, and Mogren (2010) describe a linear process from “stun to gradual balance,” indicating that it became gradually easier to live with gestational diabetes. A shifting process between two perspectives is described as “illness in foreground—wellness in foreground” (Paterson, 2001), “living a life—living an illness” (Whittemore & Dixon, 2008), and “holding on—letting go,” as taking and relinquishing control (Aujoulat, Marcolongo, Bonadiman, & Deccache, 2008). Turning points, such as critical situations or life changing events, contribute to a shifting process as the person makes a shift in meaning (Hörnsten et al., 2011; King et al., 2003). Turning points related to living with a chronic illness are further described as making people more aware of the severity of the illness and becoming more engaged in the process than earlier, which is important for their illness integration (Hörnsten et al., 2011; Meleis et al., 2000). Others describe the different phases a person goes through for coming to terms with living with illness as a fluctuating (Kralik et al., 2005; Paterson & Thorne, 2000) or a circular process (Hörnsten et al., 2011). The social context (Paterson, 2001), personal perception, and expectations of living with illness (Krichbaum, Aarestad, & Buethe, 2003) are also described as influencing the learning process.

Even if research has been conducted in this area, there are still unanswered questions about the influence the duration of an illness has on the learning process. This knowledge is important as timing and the stage in a person’s illness trajectory are important considerations that affect health care educational interventions (Chapple & Rogers, 2001). Existing studies describing patterns of management in relation to diabetes mainly address certain aspects, for example, Riegel et al. (2012) focusing on decision-making and reflection. Hörnsten et al. (2011) focused on the relationship between emotions and strategies used by those with diabetes. Studies describing the learning process when living with diabetes over time are, to the best of our knowledge, scarce and consist either of participants with a large variation in illness duration (Price, 1993) or include only participants who have been considered to have good blood glucose control (Hernandez, 1995; Paterson & Thorne, 2000). One exception is Audulv (2013) who focuses on the self-management process for persons with different chronic illnesses. Learning to live with diabetes is a lifelong process for which there is limited knowledge of how it is experienced and established over time.

## Aim

The aim of the study was to identify patterns in learning when living with diabetes, from recently being diagnosed and over a 3-year period.

## Method

### Design

An assumption in the present study was the understanding that learning takes place in everyday life and is interlaced with change, experience, development, and understanding (Jarvis et al., 2003). According to Saldaña (2003), change is interlaced with time and in order to understand change a timespan is required for reflection between “then” and “now” Based on those assumptions a longitudinal qualitative descriptive design was chosen.

### Participants and procedure

The participants were recruited from an endocrinological ward at a Swedish metropolitan university hospital. The inclusion criteria for this study were: recently being diagnosed with diabetes within the preceding 1 or 2 months, older than 18 years, and Swedish-speaking participants. About a week after being discharged from hospital after being treated for newly diagnosed diabetes, potential participants were informed about the study by mail. A few days later, they were contacted by telephone and could ask questions before deciding whether to participate. The participants were recruited consecutively at first and later on selectively with the aim of attaining a variation in sex and age. In total, 16 persons were asked to participate of whom 3 women declined. This resulted in 13 participants, women (4) and men (9), with various social (3 living alone, 10 living with partner) and working conditions (11 working, 2 not working) and different types of diabetes (five with type 1 and eight with type 2) as well as a variation in ages between 26 and 65 years (*x*¯ 44, median 43). All participants required insulin therapy initially.

### Data collection

Participants were interviewed on three different occasions after being diagnosed with diabetes; between the first and second month, after 1 year and after 3 years. All interviews had the same interview questions. The initial question was “How do you experience living with diabetes?” Later on the participants were asked to narrate situations where they had had to take their diabetes into account. In order to reach a deeper understanding the interviewer asked follow up questions such as “Could you tell me more about that?” “What did you do?,” or “What did you feel?” Interviews were conducted at the hospital, at the place of work, or in participants’ homes, according to participants’ preferences, and lasted between 45 and 70 min. All interviews were audio taped and transcribed verbatim. Because all participants (13) agreed to be interviewed at all three time-points, the analysis included 39 interviews.

### Analysis

The entire analysis process has been inspired by the thoughts of Saldaña (2003). Saldaña does not provide a method for analyzing qualitative longitudinal data but focuses on open-ended questions of a framing, analytic, and interpretive character, which are useful in describing “temporal-based themes.” As a first step, an inductive qualitative content analysis (Graneheim & Lundman, 2004) was conducted for each interview. The interviews were read through in order to grasp the content in its entirety. Meaning units were then identified, consisting of words or sentences that related to the same meaning, formulated with the purpose of shortening but preserving the core of the text (Graneheim & Lundman, 2004). For each interview, the formulated meaning units were sorted under subcategories, and then brought together into categories. A theme was formulated based on the thread seen through the subcategories and categories for each interview (Graneheim & Lundman, 2004; see Table I). This procedure is in line with Saldaña (2003) describing focusing on words summarizing the core of the text, which are compared with each other in order to discover what they share together and how they integrate with each other. Table II shows an example of themes, categories, and subcategories for one participant for the three interview occasions.

**Table I:** An example of the analytical steps from meaning units to theme (P05).

Meaning unit	Formulated meaning unit	Subcategory	Category	Theme
“Then I prioritize myself last, so it’s, it’s easy to fix everything with the job and the children, and then you sit on the couch and fall asleep and then you have missed the night insulin”	Have other priorities than one’s own needs, forgetting to take the syringe	Priority problems	Dependent	Increasingly difficult to take care of oneself

**Table II:** One participant’s themes, categories, and subcategories for the 3-year period (P05).

Theme	Diagnosis reason for lifestyle change	Aggravating circumstances give insight on own vulnerability	Increasingly difficult to take care of oneself
Category	Positive change	New vulnerability	Dependent
Subcategory	-Feeling better	-Necessary routines	-Health care crucial function
Subcategory	-Explaining diagnosis	-Lack of continuity	-Priority problems
Category	New situation	Knowledge application	Change in attitude
Subcategory	-Initiation change	-Active application	-Renegotiated illness
Subcategory	-New priority	-Involvement of others	-Difficulties
Subcategory	-Knowledge search		-Insufficient glucose levels

**Figure 1:**
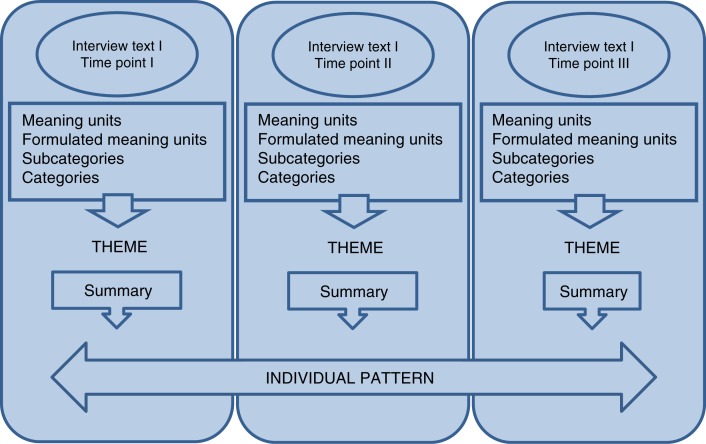
Schematic illustration of development of one individual pattern.

In the next step, a summary based on the content of the subcategories, categories, and theme for each interview was formulated. In order to be able to distinguish a pattern for each person, changes and differences concerning the person’s thoughts, feelings, and management of their illness were focused in the new texts from the different occasions. The analysis question “How can the process of learning be described for this person?” was kept in mind when formulating a description of the individual pattern. The focus of the analysis was to describe the complexity of the process instead of what Saldaña (2003) describes as a “from-to” analysis focusing on the product of the process. (see Figure 1 for the process for one participant).

As a final step, the texts describing individual patterns were compared with each other to see how they related to each other, and shared commonalities were brought together to form a main pattern. See Table III for one example of a main pattern consisting of two participants’ themes, categories, and subcategories. To ensure trustworthiness, memos and reflections were continuously written down during the analysis. The memos were used in the process when the authors compared these with the categories and themes that emerged. The analysis was also repeatedly discussed within the research group and at research seminars. Examples of quotations from the interviews are presented in the findings.

**Table III:** Development of one main pattern.

Main pattern: To find the balance gradually letting oneself to live						

	Occasion I		Occasion II		Occasion III	
			
	P10	P13	P10	P13	P10	P13
Theme	Blood glucose control important in an emotional existence	Glucose control highest priority in a changed life	Better equipped to deal with a changed life	A more balanced life where glucose control got an altered meaning	Mastery living with diabetes where a changed attitude toward glucose control is important for feeling alive	Sufficient knowledge to live life
Category (with subcategories)	Fluctuating feelings (hope, despair, uncertainty, positive elements)	Fluctuating emotions (though changes, positive conditions, insecurity, doubts, hopefulness)	Easier life (improvement, more knowledgeable, changed attitude)	Easier life (better balance, habit)	Balance (habit, easier treatment, new priorities, present blood glucose)	Diabetes part of life (normalized life, successful strategies, glucose constantly present)
	Unsatisfactory management (knowledge needs, glucose understanding, unsatisfactory choices)	Limited capacity (emotional information search, routines central)	Positive and negative changes (different changes, others’ double meaning)	Embodied knowledge (successful strategies, independence)	Control (independency, knowledge, satisfactory, management)	New challenges (another uncertainty, quiz, questions of different nature, ongoing changes)
				Others’ influence (positive encounters, manage surroundings)		

### Ethics

The study was approved by the regional research ethics committee (Dnr 03-589) and was performed in line with the Helsinki Declaration (2014). Before each new interview, participants received both written and oral information and gave their verbal informed consent. Participants were informed of their right to withdraw from the study at any time and that confidentiality of information given in interviews was guaranteed.

## Findings

Five main patterns were distinguished in the analysis and were then developed after the diagnosis and over the following 3 years.

I: To find the balance, gradually letting oneself live; II: Active searching for knowledge to gain control; III: Despite obstacles to have the strength to do what is important; IV: The hazards dominate when it becomes increasingly difficult to attain an acceptable blood glucose level; V: With knowledge and motivation adapt to minor changes in daily life. The study showed that the learning process related to living with diabetes can be both linear (Pattern I, V), shifting (Pattern II), as well fluctuating on a more daily basis (Pattern III). With time, participants in four of the patterns experienced themselves being more capable of managing a life with diabetes. Unlike other patterns, participants in Pattern IV instead found learning to live with diabetes becoming increasingly difficult with over the duration of the illness. The present study showed that the understanding of the illness and of self-management was changing and developing over time and differed for participants in different patterns.

I: To find the balance, gradually letting oneself live (P10, P13)

Initially optimal blood glucose level had highest priority, at the same time limited experience made the participants feel they had few options for action. Different learning needs, especially in relation to information, were experienced. Not to be able to do what one previously had done was experienced as a consequence of the illness, but also as the only choice when an optimal blood glucose level had to be achieved. To ensure a regular lifestyle became important, including allowing a focus on one’s own needs. Strategies to ensure desired blood glucose levels were glucose monitoring and information seeking, together with an appropriate diet and exercise, which became more important than ever.

You think a bit more about that, “I shouldn’t really have this extra sandwich,” although I am a little hungry … I never really got used to that, before I ate until I was satisfied, but I also think it is more important now to avoid taking extra insulin, so I’ll leave the sandwich, even though I am really hungry. That’s the way it works now and I think it’s probably worth it. (P10, first interview)

Later on in the learning process a change in attitude toward blood glucose levels was expressed. To allow oneself to sometimes do and eat what one wanted became important even if it affected blood glucose negatively. A change in understanding and insight emerged through sharing experiences with others or by feeling pressured by placing high demands on oneself. There was also a need to live here and now. It was a relief allowing oneself to drop the idea of constantly maintaining optimal blood glucose levels, which also made one feel less controlled by the illness. More information, gained by seeking information oneself together with experience, increased the ability to identify and understand bodily signs, and to deal with illness in different situations as a natural part of living.

In the beginning I was very stressed, studied books and really worked hard to get my food intake right, felt oh Jesus I can’t eat this or that. I set limits, but I have now eased off quite a bit, and it’s less stressful. (P13, second interview)

When satisfactory blood glucose levels no longer restricted participants in their daily living, new questions emerged, but there was uncertainty as to with whom one could discuss these questions. Health care was mainly experienced as a source of advice on medical treatment, such as insulin dosage.

A woman expressed her thoughts about pregnancy and diabetes; What would a pregnancy be like, and how difficult would it be later on to take care of a little child, and at the same time take care of my other child who is diabetic … those are the things that worry me now … and I don’t really know who to discuss it all with. (P13, third interview)

The participants in this pattern, told how their HbA1c values were within the reference range throughout the 3 years. However, fluctuating glucose levels still set demands both physically and mentally. Feeling good assumed learning how to balance between optimal blood glucose levels and feeling able to live as one wanted.

II: Active searching for knowledge to gain control (P03, P04, P11)

Participants in this pattern sought knowledge through a combination of applying the advice of health care personnel and exposing themselves more unconditionally to new situations. By holding on to previous habits, for example, in relation to working conditions or eating, participants ended up in situations they had to handle and understand when not feeling well and/or as a result of abnormal blood glucose levels. Situations were managed through active problem solving and by collaboration with health care. Reflections and glucose monitoring were important for understanding how to manage similar situations later on.

When I came home from the hospital I thought I would test what it would be like to go out and drink alcohol, and I went out and it went really well … you could say that I just carried on as normal. But I didn’t really because when I came home that evening I had something to eat. They told me at the hospital that I should eat when I got home, and that was at the back of my mind. (P03, first interview)

Although having fluctuating blood glucose levels was hard, the new situation was exciting and a positive reason for change, such as eating more regularly, and learning more about one’s own body. At the second stage of interview, participants’ emotions were mixed, as they had experienced vulnerability.

Then I had a stomach illness … I was admitted to the hospital for 4 days, and I really thought I was going to die there and then. I have never felt so ill. There’s no doubt about it, I thought, I’m not going to make it. I lay on the floor and couldn’t make it to the door, then they came and took care of me. (P03, second interview)

It appeared to be more difficult to live with diabetes than earlier and new questions in relation to management and adjusting medication were experienced, “I was unsure, Have I taken too little insulin or too much. When I get high glucose levels is it because I have missed a bodily sign?” (P04, second interview). Feelings of no longer having the same control were frustrating and created a new need for information and support from health care professionals. Later in the learning process, participants felt they once again recaptured control of the illness and self-management was a natural part of living. Through a range of experiences, both positive and negative, they had obtained a range of knowledge of how blood glucose was affected and of strategies to manage different situations. Additional blood glucose tests, prioritizing regularity in lifestyle, as well as learning from others were the strategies used. To be in control was to have freedom to do what one wanted, including being able to choose glucose levels.

One participant told how he handled the situation on long car drives;

Well, to avoid stopping in the middle of everything, eating and injecting in the car, I thought it’s probably better to drive with a blood sugar level around 10(mmol/l), instead of trying to get it hovering around the 5/6(mmol/l) mark … better to leave it high for now and then perhaps it will be about normal when I arrive instead. (P03, third interview)

Participants recognized themselves changed as a person. Learning about themselves including their own bodies, was seen as an advantage as well as providing new insights and priorities in life.

A summary of Patterns I and II shows that participants have common features in their processes. Participants were active in their learning process and after 3 years self-management was integrated into daily living, including being changed as a person. What differs between Patterns I and II is the trajectory from uncertainty to achieved control. In order to obtain control, participants in Pattern I made limitations in daily life while participants in Pattern II exposed themselves to problematic situations. Both aimed to manage a new life situation, but with longer illness duration becoming too demanding a redefinition of priorities and management became necessary (I, II).

III: Despite obstacles, having the strength to do what is important (P07, P12)

Soon after the diagnosis of diabetes, participants in this pattern got to know that they probably had had the disease for some time, already causing complications such as severe sight loss or neurogenic pain. The changed body was no longer reliable and rapidly constituted a major obstacle to daily living, described as “being caught unawares” (P07 first interview). Keeping blood glucose under control became a secondary priority in a chaotic existence. For the participants making lifestyle changes felt impossible in a situation where the demands to manage complications occupied their whole existence. “I was so engrossed in the pain that I wasn’t really taking in that I would have to take insulin for the rest of my life” (P07, first interview).

By the time of the second data collection life had altered in a negative way. Not being able to work or participate in social life made participants feel different, which was experienced as sad. A redefinition of what health care could provide was undertaken. Even if just hoping for medical treatment that could make life easier, participants that earlier trusted health care to solve all medical problems now experienced this to be of limited help. “You go to the doctor when you are ill and you think that you will get treatment, and then you will get better and be well again, but now I understand that it is not quite like that” (P12, second interview).

At the third data collection participants told that life had become more manageable despite losses. In order to move on in the process it was important to accept and adapt to a changed body that no longer permitted them to do what they wanted. Reflection became important in focusing on what activities to prioritize and what to accept was no longer manageable. To ensure optimum use of the body’s current capacity, planning was important to enable the best available choices.

At the same time obstacles, such as the side effects of pharmaceutical treatment, bodily impairment, and facing tedious rules, constantly arose. In order to overcome these obstacles it was essential to be active.

A man described the rules for maternity leave that did not suit his family;

She (my wife) had taken parental leave when I became so ill, and the idea was that I would then take parental leave … but that didn’t work because I was so ill that we couldn’t guarantee that on any given day I would be able to look after the children. So she carried on staying at home even after we had gone over the limit for parental leave, and the money just began to disappear. (P07, third interview)

Participants described how they found practical solutions in everyday life, asserted their rights with employers and benefits, and sought alternatives within the health and complementary care systems when they were not pleased with the care provided. Three years after diagnosis life started to become more in balance, and an interest in making lifestyle changes emerged because this seemed more manageable.

IV: The hazards dominate when it becomes increasingly difficult to attain an acceptable blood glucose level (P01, P05, P08)

Because of dissatisfaction with an earlier lifestyle, illness was initially perceived as a positive reason for considering lifestyle changes. The diagnosis also meant medical help made participants feel better than earlier. Initially, living with diabetes was manageable and the participants expressed not having specific learning needs as they knew what they had to do, because they had received information about healthy food and physical activity. At the same time, there was uncertainty about the seriousness of the illness.

So it’s possible to work out how the blood glucose will be affected when you are going to do some physical work at the weekend … you know you will need a little less insulin, but I just take the same dose of insulin and eat even more, you always need more food if you are going to do physical work. (P01, first interview)

Gradually, the participants tried to apply some lifestyle changes, but found it difficult. Learning needs in relation to planning and practical management were raised. Participants found themselves dependent on routines for self-management but did not succeed in maintaining these. Even if they knew what to do it was too difficult to put it into practice.

I feel better when I have taken the medicine, I feel really well, but if I don’t bother to take it I get tired, my eyes hurt and I get headaches. It makes such a difference and I realise I really should do as I have been told. (P05, second interview)

At the third data collection, participants expressed resignation as they experienced that, whatever they did, blood glucose levels never improved. Participants became passive even if it also meant feeling bad both bodily and psychologically. Although there was a desire for change, the obstacles to do so, such as experiencing lack of knowledge, time, or energy, hampered the learning process. At the same time, new learning needs were experienced as increased uncertainty about one’s own needs or overriding needs made it difficult to know how to act. Participants felt dependent on health care for advice on how to manage and adjust to diabetes treatment.

It always much better when I see the doctor, when I can see the results and everything on paper … you think that you are on top of things and can manage everything, and that things are OK, but there is so much to take in. (P05, third interview)

Participants expressed not having the resources available for living well with diabetes such as getting the care needed. They found it difficult to stay in contact with health care services because of practical difficulties. It was perceived negatively to be expected to seek help in primary healthcare rather than in hospital, which was the case directly after diagnosis.

It was common for participants in Patterns III and IV to experience learning and living with diabetes as an ongoing struggle where barriers discouraged the desired way of life and being the person one wanted to be. In Pattern III, the participants became more active in their learning process and gradually felt more capable of managing life as their illness continued even if the changed body hampered the desired life. Participants in Pattern IV instead experienced increasing difficulty in participating in their own learning process as well as feeling well.

V: With knowledge and motivation adapt to minor changes in daily life (P02, P06, P09)

Initially, participants in this pattern perceived themselves as having a good basis for living well with diabetes through being knowledgeable and having pre-existing habits of healthy diet and exercise. Diabetes was experienced as a common disability that everyone was familiar with “most people you talk to know someone who has diabetes, and they therefore have an idea of what it’s all about” (P02, first interview). At the second data collection, learning was described in terms of adjustments in relation to blood glucose levels and being a part of life, and feeling good in general. Being able to influence the illness and be in a positive context, including favorable cooperation with health care, were perceived as facilitating adjustment. Participants had identified barriers that hampered achieving a satisfactory blood glucose level, such as maintaining a healthy diet or remembering medication, but also found effective strategies to address these obstacles, “a good way of managing is to think about something else, or to quickly leave the kitchen at work and start working on something, to give yourself something else to think about other than that you would like something sweet to eat” (P09, second interview).

Later on in the learning process, participants had found a balance in food that was both healthy and tasty. Experiencing eating more healthily than before and healthier than others was positive “you need to be aware of what you eat and not just sit at the table shovel in everything on offer without thinking about it” (P02, third interview). Self-management was more difficult, although still manageable, when other illnesses occurred or daily life conditions changed. The participants described blood glucose levels oscillating between normal to slightly elevated levels over the 3-year period. For participants in this pattern, learning in relation to living with diabetes was mainly about adapting to different food and medication, something that required knowledge but within a limited area, and was considered as just an additional element in life. This differs from the participants in the other patterns as they experienced a more transformative learning process making a greater impact on them as a person, their health, and life.

## Discussion

The present study shows that, despite the same duration of illness, participants in the different patterns had different learning needs in relation to living with diabetes. The type of nursing intervention a person with diabetes needs is thereby not determined by illness duration. In the present study, the change in understanding of the illness as well as of self-management did not occur as a single event, as a turning point (King et al., 2003), instead it was more a gradual change resulting from several events that together contributed to a new understanding. The changed understanding could mean a decision to give illness and self-management less space and attention than earlier (seen in Pattern I); to allow oneself to live more as one wanted, a more normal life, described by Jutterström, Isaksson, Sandström, and Hörnsten (2012) as hoping for a more balanced existence. In differ from experience it more difficult to manage illness than earlier (Pattern II), followed by an increased involvement and priority of self-management in line with Hernandez (1995) and Jutterström et al. (2012), both describing the turning point, a life changing event, as “being at a crossroads with no return” (p. 3). Finding illness more difficult and severe to live with than expected could in the present study also mean becoming less active in the learning process (Pattern IV).

In the present study, as well as in other studies (e.g., Van Dam, Van der Horst, Van den Borne, Ryckman, & Crebolder, 2003; Vég, 2006), being active was found to be essential for moving forward in one’s learning process. To be active was to use different ways or strategies to reflect and seek and read information, to be more knowledgeable, and find pragmatic solutions facilitating everyday life, findings which are in line with Jarvis et al. (2003). This is further illuminated by Ekebergh (2007) describing reflection and learning as being strongly linked together, with reflection making situations and activities available for analysis and understanding. Interlaced with reflection was an increased consciousness of a changed body. There was a changing ability for body listening between the patterns as well as over time. To recognize signs and to understand associations between the body’s responses in different situations was important for learning. Others have described body listening as important for illness integration and successful self-management in achievement of satisfactory blood glucose control (Kralik et al., 2004; Paterson & Thorne, 2000; Paterson et al., 1998; Price, 1993; Thorne, Paterson, & Russel, 2003). It is worth considering how participants in Pattern II had the ability immediately after diagnosis to start investigating different situations by exploring, body listening, evaluation, and reflection. This differs from Price (1993) describing a “trial and error phase,” as something a person does later on in the learning process after first strictly following health care advice described as a “getting regulated phase” (Price, 1993) or as “passive compliance” (Paterson & Thorne, 2000), as seen in Pattern I. At the same time, participants in Pattern II experienced that learning is not easy, instead it involves a lot of effort and also includes making painful mistakes, described by Hill-Briggs (2003) as essential for the management of daily barriers when living with diabetes. In the present study, knowledge development was, to a different extent, a result of a mutual interdependency between experienced based learning with theoretical knowledge and reflection. This interaction between different ways of learning ending up in an interweaving of personal knowledge, also described in terms of embodied knowledge or integrated knowledge (Bullington, 2013), is important for feeling in control and being capable of living well with illness (Hernandez, 1995). Even if learning strategies used by participants in the present study had much in common with general learning strategies (Jarvis et al., 2003), learning to live with diabetes was different. Learning was “forced” on the participants, especially seen in Pattern III, as learning took place in an existence dominated by struggle, suffering, and losses.

Pattern IV differed considerably from the other patterns as participants over time gradually felt living with diabetes became more difficult. The use of learning strategies in Pattern IV was also different from the participants in the other patterns. The constant testing and evaluation of different actions in daily life, seen in the other patterns, was not as common here. Even if participants in Pattern IV wished for changes in order to feel well, unsurmountable obstacles inhibited their ability to effect those changes. Whittemore and Dixon (2008) highlight obstacles hampering a person’s ability to move forward in a learning process, described by Kralik (2002) as staying in a phase of turmoil and distress. Participants in Pattern IV also found it difficult to reflect, making it difficult to identify options and to predict what was going to happen, which inhibited their ability to prioritize as well as to plan. Spenceley and Williams (2006) found that not being able to plan and prioritize were associated with dissatisfaction in relation to self-management. Even if participants in all patterns faced obstacles, obstacles for participants in Pattern IV were more something that “hit” the person, as it was not expected, and decreased their chances of finding strategies to either avoid or manage obstacles. Another difference between participants in Pattern IV and the other patterns was their interpretation of bodily signs. For participants in Pattern IV, a changed body was salient but there was insecurity in relation to understanding those signs. A feeling of not being able to manage situations, together with more or less constantly having unsatisfactory blood glucose levels, were frustrating, which can be understood in terms of disharmony (Merleau-Ponty, 1945/2002) or lack of change in potential related to new demands (Vygotsky, 1978).

### Methodological considerations

The greatest advantage with the present study was its longitudinal approach, which allowed the identification of patterns over time. The fact that all participants remained in the study for all 3 years made it possible to analyse individual processes and patterns, increasing the credibility of the study as the individual patterns formed the foundation for describing the main patterns. The longitudinal design was inspired by Saldaña (2003) who claims that if a process is of interest, repeated data collection over time is needed even if it is time-consuming. By focusing on the process of learning, specific events or other specific influencing factors have not been taken into consideration. Instead patterns were developed inductively, based on similarities regarding how participants understood and managed their changed life situation with a diabetes diagnosis. It was surprising how similar the process was for participants that together conducted a shared pattern. Only one participant (P05) was considered to not only belong to Pattern IV but also to have much in common with the Pattern III description. However, it would be interesting to research further to see if other people with diabetes could be recognized within these described patterns, and to see if persons switch between patterns over time or stay within a pattern. In order to reach a rich variation of the phenomena of interest, participants of varying sex and age were selected (Graneheim & Lundman, 2004). Even if a selective approach was conducted later on in the recruiting process there were still more men than women included in the study. This was because of three invited women declining the offer to participate. Contexts, such as the social and health care systems, are of importance for a person’s learning process (Dubouloz et al., 2010). One has to bear in mind that all participants came from the same geographical area and were recruited from a hospital setting; this is a limitation when transferring findings from this study to other contexts. It is also worth considering that 3 years is a short period in the trajectory of someone with a chronic illness and with an extended period of time for data collection the patterns probably would have been described differently. Asking for specific situations in daily life in the interviews was inspired by Price (1993) and Thorne et al. (2003) who concentrated on situations in everyday life when illuminating learning. To increase the trustworthiness of the analysis, illustrations of different parts of the process has been presented in the method section. Also, quotations from the transcribed text have illustrated the findings in line with Graneheim and Lundman’s (2004) recommendations. The findings have been discussed with different researchers in order to stay open for a varying understanding of the text (Saldaña, 2003), and to clarify the process of analysis and formulation that were perceived as unclear or difficult to understand, in order to reach consensus about interpretation. In the present study, type of diabetes was not considered of main importance, because the focus was on experience related to a changed health situation and new demands after being diagnosed with diabetes. In diabetes research otherwise it is common to select participants based on type of diabetes. Different types of diabetes as well as different medical regimes were considered in the present study to give a desired variety of experience in relation to the learning process.

### Clinical implications

Because, despite the same duration of illness, participants in different patterns showed differences in learning needs as well as learning strategies, health care needs to be flexible enough to facilitate learning. To be able to achieve this, personalized nursing interventions based on patients’ needs, beliefs, and circumstances have to be carried out, as described by McCormack and McCance (2006) in their model for person-centered care. In line with findings from this study, patient involvement in decision-making and goal setting is essential in person-centered care, as it is not possible to predict a person’s learning needs. Person-centered education has also been shown to contribute toward success in achieving positive and lasting change for improving a patient’s knowledge of their health, improving their ability to live with illness, improving metabolic control, and providing greater satisfaction with care (Coulter & Ellins, 2006; Hörnsten, Stenlund, Lundman, & Sandström, 2008). However, research shows that the pedagogical approach based on formalized rather than problem-oriented procedures is still often adopted (Thors Adolfsson, 2008). To be able to recognize individual needs and to facilitate learning, nurses need to have knowledge of the variety of experience related to learning to live with diabetes as experienced by those living with the illness.

## Conclusion

A longitudinal design following participants over a 3-year period made it possible to describe the development of five different learning patterns in relation to living with diabetes. It was shown that even if participants had the same duration of illness, participants in different patterns had different learning processes and that their need for health care assistance differed between participants between patterns and over time. A person-centered care is needed to meet the different and changing needs of those living with diabetes as they learn to live with a lifelong illness.
